# Applying Mobile Augmented Reality (AR) to Teach Interior Design Students in Layout Plans: Evaluation of Learning Effectiveness Based on the ARCS Model of Learning Motivation Theory

**DOI:** 10.3390/s20010105

**Published:** 2019-12-23

**Authors:** Yuh-Shihng Chang, Kuo-Jui Hu, Cheng-Wei Chiang, Artur Lugmayr

**Affiliations:** 1 Department of Information Management, National Chin-Yi University of Technology, No.57, Sec. 2, Zhongshan Rd., Taiping Dist., Taichung 41170, Taiwan; eric_chang@ncut.edu.tw; 2 Department of Inter Design, Asia University, Taichung 413, Taiwan; 3 Graduate Institute of Color & Illumination Technology, National Taiwan University of Science and Technology, No.43, Sec.4, Keelung Rd., Da’an Dist., Taipei 10607, Taiwan; kuojui@mail.ntust.edu.tw; 4 Department of Digital Content Design, Ling Tung University, No.1, Ling tung Rd., Taichung 408, Taiwan; 5 Edith Cowan University (ECU), 6000 Perth, Australia; artur.lugmayr@artur-lugmayr.com; 6 Aalto University, 2150 Helsinki, Finland

**Keywords:** mobile augmented reality, mLearning, interior design, Augmented Reality (AR), Attention, Relevance, Confidence, and Satisfaction (ARCS), learning motivation, ARCS, learning design

## Abstract

In this paper we present a mobile augmented reality (MAR) application supporting teaching activities in interior design. The application supports students in learning interior layout design, interior design symbols, and the effects of different design layout decisions. Utilizing the latest AR technology, users can place 3D models of virtual objects as e.g., chairs or tables on top of a design layout plan and interact with these on their mobile devices. Students can experience alternative design decision in real-time and increases the special perception of interior designs. Our system fully supports the import of interior deployment layouts and the generation of 3D models based on design artefacts based on typical design layout plan design symbols and allows the user to investigate different design alternatives. We applied John Keller’s Attention, Relevance, Confidence, and Satisfaction (ARCS) learning motivation model to validate our solution to examine the students’ willingness and verify the ability of students to improve learning through MAR technology. We compared a sample experimental group of *N* = 52 test-subjects with a sample of *N* = 48 candidates in a control group. Learning indicators as learning interest, confidence, satisfaction and effective have been utilized to assess the students’ learning motivation through the use of MAR technology. The learning results have been determined by the independent sample *t* testing. The significance of the post-test had a *p*-value < 0.05 difference. The result of the study clearly shows that the reference group utilizing MAR technology as a learning aid show a higher learning effectiveness as the control group. Thus, we conclude that MAR technology does enhance students’ learning ability for interior design and making appropriate design decisions.

## 1. Introduction

The use of digital media supporting learning and integrating these into traditional teaching methods has become mainstream of today’s learning designs [1,2,3]. In particular, applying augmented reality (AR) in education is becoming increasingly popular, as it provides an interactive learning experience. It also allows embedding artificial computer-generated artefacts throughout the ‘real world’, thus allowing students to experience learning content in the real world, rather than a 2D-based system [4]. AR technology, in contrast to ubiquitous computation [5], mixes computer generated virtual worlds with real-world scenes and creates imagery combining virtual and real worlds. AR enables also amazing new interaction possibilities with the newly created world. The prevalence of smart phones in terms of consumer penetration, makes these a platform of choice for AR applications. Their application in educational settings allows a sense of presence and is fun to use for students. The positive effect, value, the students’ pleasure of utilizing AR, and the enhancement of students’ attention has been confirmed in today’s learning literature, as in for example [6].

We all know how difficult it is for people to imagine and understand 2D interior layout plans without seeing the result in the real world or through a 3D simulation. Hence, AR technology seems today the most suitable candidate to overcome this issue and has been continuously proposed for interior design applications by several authors. To pick one example [7], uses a cell phone or tablets to place and augment furniture interior spaces. These devices are not commonly available for students to learn deployment layouts, interior design symbols, or the evaluation of alternative design layouts. In this paper we present our application of mobile augmented reality (MAR) technology for learning interior design students. Our solution takes advantage of the dramatic progress of digital AR technology, and we rely solely on a tracking marker that is placed onto the deployment layout to define the interior space and the interior design symbols in the room. Next, our system renders the complete 3D model of virtual furniture on the mobile phone screen.

### 1.1. The Importance of Motivation in Learning

From the definition point of view, the meaning of ‘to be motivated’ relates to be ‘moved to do something’. Thus, when someone who is ‘energized’ or ‘activated’, he can be considered as ‘motivated’ [8,9]. This is related to optimal learning outcomes. To be motivated in learning is highly correlated with learning effectiveness. A strong motivation allows people to focus on tasks for a long time, and easily being immersed into the flow of experience. Factors underlying motivation are attitudes and goals giving raise to action—it concerns the explanation (the ‘why?’) of actions underlying the motivation. As pointed out by Ryan and Deci in [10], the orientation of motivation concerns the underlying motivation can be dived into intrinsic and extrinsic types. Both have an important impact on learning. Stating an example, a student can be highly motivated to do homework out of curiosity and interest—or alternatively because he or she wants to procure the approval of a teacher or parent. A student could be motivated to learn a new set of skills because he or she understands their potential utility or value—or alternatively because learning the skills will yield a good grade and the privileges a good grade offers [10]. Much internal motivation is driven by external motivation. For example, the content of the textbook itself is interesting and thus arouses the interest of learners’ internal learning and the motivation of active learning.

The performance created through the learning process refers to the benefits created by the learner’s internal and external motivations. Motivation impacts students’ behavior, and with increasing motivation the learning performs increases. Both personal and environmental factors (input facets) influence the level of effort, behavior performance, and teach outcomes (output facets) that learners are willing to take. The more efforts learners undertake, the better they will perform. The better results learners achieve, the stronger the motivation to continue with efforts to achieve even better results. This phenomenon is cased the “virtuous circle of learning” [11].

### 1.2. Keller’s Attention–Relevance–Confidence–Satisfaction (ARCS) Motivational Learning Design Theory

John Keller proposed the Attention–Relevance–Confidence–Satisfaction (ARCS) motivation design model in 1987 [12,13,14,15], which was divided into four factors relevant to improve the learning effectiveness of students. ARCS emphasizes that the motivation of learners must be matched with the use of these four factors in order to improve students’ learning performance. Instructional design and improvement of teaching materials are the most important factors that determine students’ motivation and interest in learning. Good teaching content design can arouse students’ attention and interest, let learners have confidence in the topics and content of learning, help students build their own learning ability, and finally allow students gain satisfaction after learning. The ARCS model can be used to verify whether the design of teaching materials effectively stimulates students’ motivation and learning effectiveness [12,13,14,15]. In this study we want to confirm whether AR technology as a teaching medium can stimulate learning confidence and effectively improve students’ learning satisfaction and learning results. In this study, experimental teaching combined with statistical verification and the integration of MAR technology into interior design courses can enhance students’ learning interest.

According to Keller’s [12] research on motivation theory in learning psychology, teaching and learning processes can be divided into two major facets: input and output. The input facets include personal factors and environmental factors; where the output facet is the learner’s effort, performance, and learning outcomes. Personal factors include learning motivation, interest in learning, personal learning ability, knowledge, and skills already possessed. Environmental factors include the strengthening of learning motivation, teaching design, and management of teaching methods. That is, when students pay attention to study, they will be influenced by factors such as interesting content, learning mood and environmental atmosphere [12].

### 1.3. Converging Keller’s ARCS Motivational Learning Design Theory with New Technologies such as Augmented Reality (AR)

**Figure 1:**
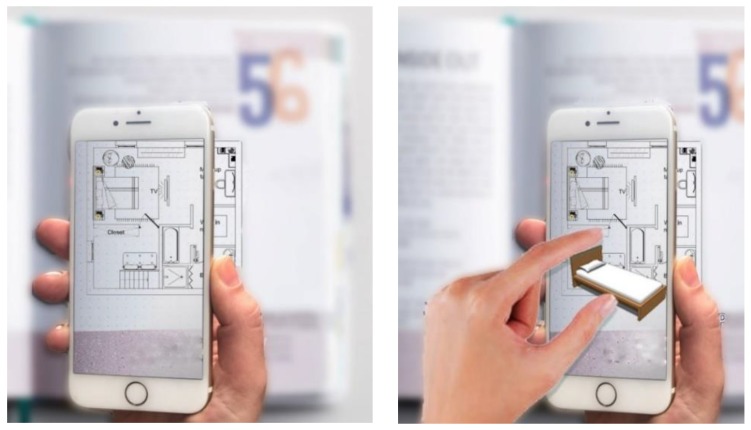
Overview of our mobile augmented reality (MAR)-based teaching tool to support students in the learning process of interior design.

Although AR is a well-integrated technology in teaching research, and most studies of students using AR show improved learning performance, we would like to investigate the true factors that drive learning effectiveness on the example of our developed system. We intend to discover whether learners appreciate AR based learning methods and focus on factors as e.g., learning motivation, learning interests, learning attitudes, and changes in instructional. As the main theory of this study is based on the ARCS motivation theory proposed by Keller [13], we conducted a teaching experiment to verify that integrating MAR as teaching material into instructional design can significantly improve the students’ learning outcomes in interior design course. We also aimed at investigating, weather design knowledge and understanding improves through AR-based solutions, in terms of increased learning efficiency, in particular in envisioning the final design outcomes, and plan interior design spaces. Keller’s work in motivational instructional design theories (see [12,13,14,15]) clearly emphasizes the tight relation between learning motivation, teaching, design, and learning performance by integrating research results of psychology related motivational theory with teaching design models. It also provides an excellent review of general valid teaching models that can be used in the classroom. An overview is given in Figure 1.

### 1.4. Aims and Goals of this Study

Let’s focus firstly on the study underlying research questions. We aim at answering the following research questions:

- Are Keller’s theories in motivational instructional design theories applicable for new digital learning technologies such as MAR?
- In which ways does MAR-based technology improve the various components of Keller’s ARCS motivational theory consisting of Attention, Relevance, Confidence, and Satisfaction?
- Which methods are suitable to measure and verify Keller’s components in an experimental setting, utilizing new innovative technologies?
- How does MAR technology-based teaching design affect students’ motivation, interest in learning, learning satisfaction, learning outcomes, and behavior?
- In which ways can the design of interior design courses benefit from AR-based textbooks, and how does the way of learning change?

To answer this question, we have developed an AR 3D image information display interface on a mobile device as experimental teaching tool. We equipped it with AR technology, to instantly display the spatial composition of an interior design including 3D artefacts as e.g., furniture accessories. Our MAR teaching system simulates a teaching situation and incorporates different interior designs and themed content to support learning tasks. It can be used for creating interior design layouts (residential graphic design) and fitting of furniture. It has a 3D visual presentation of indoor design layouts, as well as it has indoor space setting as e.g., ceiling, floor, wall, furniture design, decoration design, orientation, lighting as a learning aid, etc.

To be able to quantify our research findings, we conducted a statistical analysis and compared two groups of students: one group being trained with traditional teaching methods (control group), and another being exposed to MAR-based learning (test- or experimental teaching group). The number of samples in this study’s experimental teaching group was N = 52, while the sample size in the traditional teaching control group was N = 48. We first assessed whether there is a significant difference in learning performance between both groups. We wanted to test, if the experimental group’s final learning effect is better than that of the control group. This would indicate that the use of MAR teaching material is an important influencing factor for learning outcomes. We further use the ARCS model as a research model to conduct regression analysis on the variables learning interest, learning attitude, confidence, and satisfaction based on the evaluation of the test group utilizing MAR teaching materials. We want to understand whether students’ attitudes towards the use of MAR teaching materials is affecting learning interest, confidence, satisfaction, and effectiveness. This is the main reason why we utilize the ARCS theoretical framework to explain to what extent MAR teaching materials affect learning.

## 2. Literature Review

### 2.1. Augmented Reality (AR)

AR has been proposed by scholars such as Azuma [16] since the 1997s, and the concept of AR system design theory went on to become mature and complete by now. Many companies and research institutes actively developed applications and research in various fields. More recently, AR technology with mobile devices and digital media in combination with a wide variety of partially commercially available applications of AR education, entertainment, shopping, medical, military, or museum navigation show the unpredictable prospects of AR. AR is a concept of mixing the “real world” with a “virtual world” overlay. AR technology superimposes information into the real world through a virtual scene. In virtual reality (VR) (systems as [17]), the consumer is immersed into a completely artificial world—AR overcomes this shortcoming, and embeds virtual content into the real world. AR has been used in the game industry, film industry, architectural design, industrial design, virtual navigation, digital learning, sports, and digital content presentation (see e.g., [18]). The use of AR in classrooms has been discussed in [19]. It not only overlays the 3D image of the virtual object on the real scene, but also create the perceived characteristics of the virtual and the real. Moreover, it allows the 3D image to be presented in the computer from any angle to achieve a more realistic experience. The newly created ‘reality’ combines virtual scenes with reality to a higher extend [8,20,21,22].

In addition, the user’s view of the real world through e.g., a translucent display merging real-world with artificial 3D imagery creates a plausible new view of the world and completely new experiences and perceptions of the real physical world. It allows users not only see the real world merged with elements of the virtual world, but also creates many amazing possibilities for interactions. Sometimes AR is referred to with the more general term mixed reality (MR). MR refers to a multi-axis spectrum of areas that cover VR, AR, telepresence, and similar kinds of technologies, in contrast to ubiquitous computational systems, which blend into the natural environment (e.g., [23] or [24]).

Obviously, the combination of AR applications and mobile devices creates more creative instructional designs in education: real-time spatial calculus, 3D stereoscopic presentation, interactive audio, video content, message sharing, object tagging, and intelligent agents. All of these novel services surpass previous digital learning software which are mostly situated in the 2D space presented through 2D screens. MAR extends the AR paradigm through the addition of mobility, utilizing the screen of a mobile device rather than see-through or head-mounted displays. MAR allows to display digital information in the user’s field-of-view through the mobile phone display, and lets computer generated objects virtually appear in the real physical world by displaying digital information in the user’s field of view on the mobile’s display screen. Both, real physical world, and digital world are rendered virtually in the same space [25]. AR is often understood as part of a MR continuum, with a focus on augmenting the real world [16]. These aspects of AR can help students to learn and understand the cultural environment to provide a rich and unique interaction experience.

More recently, AR technology is considered as a new design approach for architecture applications. As a result, a lot of AR experiments and research have been directed toward the architectural design process [26,27]. AR as a next-generation interface provides a different way of interaction with digital information. This new way to interact can be used to design better learning experiences. The definition of AR by Azuma (see [16]) is relaxed to accommodate more prototypes that could help us understand how AR can be used for education.

### 2.2. Learning Motivation Theory: The ARCS Model

ARCS is based on Keller’s systematic research approach, a design pattern of his motivation to stimulate students’ learning motivation, and integrates motivation patterns proposed by motivation theory, and related theories. In contrast to other methods as e.g., design thinking [28], he believes, that textbooks not following any kinds of instructional designs, will not be attractive or in the focus of learners, and their learning effects will be greatly reduced. Therefore, Keller [14] proposed the ARCS motivation model, which can provide educators with the motivational needs of students and identifies the design strategies for teaching, stimulates learning motivation, and effectively improves students’ learning and performance.

Four elements are proposed by Keller [14], which can help the instructor to motivate and maintain the learner’s motivation for learning. The aim of ARCS is to help in the curriculum design or in improving teaching. This model emphasises the motivation of the learner, which must be matched with the use of these four elements to achieve increased stimulation of student learning.

ARCS integrates many motivational theories, whose main goal is to strengthen the systematic teaching design, that the design of teaching materials can meet the participation and interaction of the inspiring learners. Teaching design should also provide the application of theoretical, organizational and practical aspects of teaching. The four factors A, R, C, and S are interlocking—they affect the teaching effects of teachers. Teachers should utilize several ARCS factors in their teaching at the same time, which allows students to learn following a benign cycle. The lack of mixing the different facets of the ARCS model, will greatly reduce the overall teaching effect.

Keller emphasizes that ARCS has also a ‘diagnostic’ nature and a prescribing function, which means that if learners are lacking in one or more of the ARCS aspects, there are ways to overcome these, and integrate ways into the learning design in particular for these learners. The instructor can apply a systematic teaching strategy to their inadequacies in order to improve the motivation of students to improve their learning. The four elements and definitions of ARCS proposed by Keller and the purpose of integrating the interior design curriculum of this study are illustrated in Table 1. The table also provides a solution of how ARCS can be adapted in an experimental setting to measure and verify the four components of the model through concrete variables.

According to the ARCS motivation model [14], the four elements define the application purpose, and classify the tools used in realizing the vision into four constructs, namely: 1. learning materials; 2. learning attitude; 3. learning platform; and 4. self-learning. These are the main four constructs to create appropriate learning experiences and can be utilized to verify the motivational factors of the learning design.

## 3. Mobile Augmented Reality (MAR) System

AR based interior design layouts introduce predetermined spaces in virtual layouts, which would show up as a 3D overlay over the real physical world. AR based designs also include additional augmented information and lets interior design symbols appear on e.g., a mobile screen. Our AR software is capable of creating AR models out of interior design symbols as e.g., chairs, tables, or similar artefacts.

**Figure 2:**
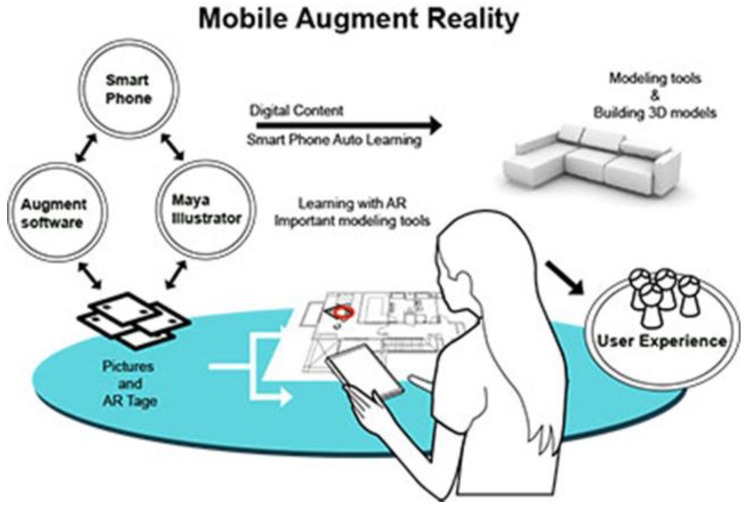
Overview of our system solution. Based on an interior layout plan, we create an augmented reality (AR) scene on mobile devices. Interior design symbols as e.g., chairs, tables, couches are converted to augmented artefacts and overlay the physical world.

The user-interface of our AR system was constructed based on our developed software tool. The software generates 3D objects based on the interior design plan, it’s interior design symbols, and then rendered in real-time as overlay of the physical world. We utilized Maya and Illustrator as tools for creating interior plans, and associated 3D models, which are converted into the correct format for AR real-time rendering. The overall process is presented in Figure 2.

### 3.1. Software Environment

To conduct this research, we applied latest AR technology to develop our system. We developed the system for smart phones and tablets, to be able to expose it to a broad audience to enjoy this interactive experience. We developed the basic software for the purpose of this research work and provided it to interior design students to improve their learning experience. The overall system architecture is similar to other Unity-based solutions, as e.g., described in [29] or [30]. This section also compares traditional learning methods with our new AR-based software environment, which is depicted in Table 2. These software that have been utilized to develop the application are enlisted below:

- Unity 2018.1.5f1: main software platform of this AR application, upon which we included the built-in Vuforia Augmented Reality Software Development Kit.
- Android SDK Tools: to export Android application package (APK) that required Kit of Unity.
- Java SE Development Kit 8: to export APK Required Kit of Unity.
- Various platforms are supported by changing the build target of our current project to e.g., iPhone operating system (iOS).
- Photoshop and Illustrator: user interface software for content creation.
- Android operating system or iOS: the platform of the MAR application.

From a user-centered software design perspective, our newly developed system aligns with the various learning steps that are typically utilized as part of traditional teaching methods, which is described in further detail in Table 2. To accomplish this, we (1) have been editing the user-interface components of the system in Unity; (2) build the 3D scene models of interior design objects; (3) developed interactivity in Unity through script development; (4) built the AR recognition system, and created markers to provide successful recognition of layout plans; (5) implemented interaction models to enable the user to interact with 3D objects; (6) and tested the application prior release. The application was exported for different target platforms, as e.g., Android Operating System or iOS.

### 3.2. Interior Deployment Layout and Symbols

**Figure 3:**
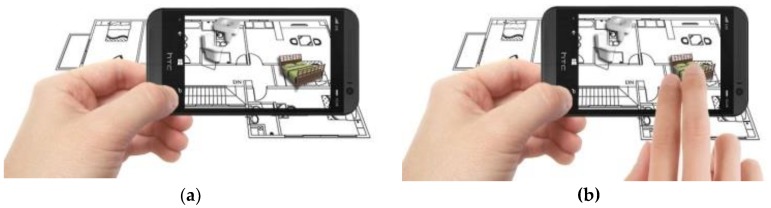
(**a**) AR-based simulation of 2D interior design layout plans and integration of 3D images of interior design symbols; (**b**) Students can evaluate different design decisions by moving 3D objects in AR on their mobile phone screens.

Multiple AR markers (or tags), which can be recognized by the camera were placed onto the 2D interior scenes, which assisted in the creation of the 3D models on top of the interior plans. As soon as these tags or markers appeared within the range of the camera, students saw the 3D augmented images of the layout design plans. This helped students to understand the structure of the interior space (see Figure 3a) more extensively. Students were able to move the mobile over the 2D interior layout plans, compare different design decisions, and learn the symbols utilized in interior design. Different design decisions can be evaluated by moving different objects of the interior design to different locations on the design layout, to evaluate design alternatives, as illustrated in Figure 3b.

## 4. Research Design, Methods and Approach

The utilization of the MAR application is incorporated into the curriculum design of interior design students, and we evaluated the effectiveness of student learning. To test learning effectiveness, we divided different users into an experimental-group using our new system, and a reference-group learning design symbols through traditional methods. We investigated the learning effectiveness and knowledge of students after and before using the system to understand the impact of utilizing this new technology on student learning outcomes. For several statistical analysis we have been utilizing IBM’s Statistical Product and Services Solution (SPSS) V.25.

### 4.1. Research Phases

**Figure 4:**
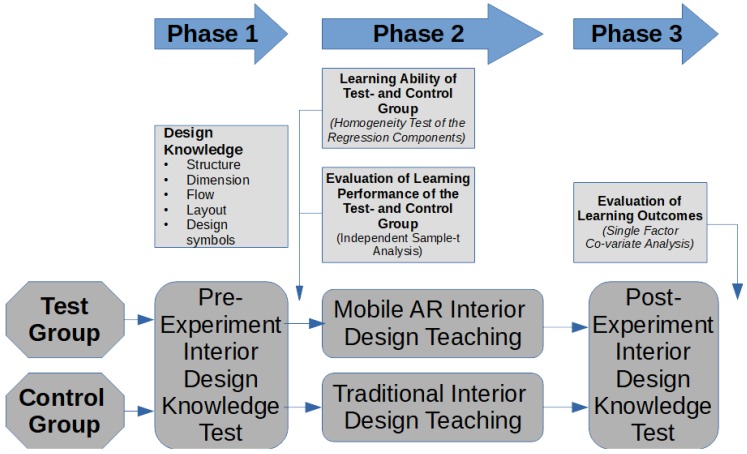
The three stages (phases) of our experiment.

The overall research approach is presented in Figure 4. Different variables, control variables, and experiments are evaluated for both user-groups through a self-evaluation in stage 1, followed by a post-experiment evaluation in stage 3 of the study. The experimental group was exposed to an AR-based teaching method in stage 2, while the control group was presented with a traditional way of teaching. The experiment was carried out in three phases (see Figure 4):

- *phase 1 (pre-test stage):* this phase was carried out one week before the experimental teaching, and both—the experimental, and the reference group were tested for their knowledge in interior design;
- *phase 2 (experimental teaching stage):* during this phase, the experimental group received two lessons (each of 40 min duration) of interior design teaching activities. We integrated our own developed mobile based AR design system for this group. The control group was exposed to the same teaching activities, however, we utilized traditional teaching activities to be able to compare learning effect;
- *phase 3 (post-experimental teaching stage):* both groups—the experimental and reference group—Have been tested after the 18th teaching week to identify the learning effectiveness, and the results of our experimental way of teaching.

We tested variables such as pre-test scores for both test groups, and the execution of the experimental teaching by educators. To verify our experiment, we imposed some restrictions in our experimental design: first, both groups were taught by the same teacher, to exclude factors of different ways and cultures of teaching; second, both test groups were subject to pre- and post-testing; and thirdly, the same teacher was responsible to score the students’ outcome of alternative design decisions, and students’ learning effect.

Our test-groups were composed of more than 100 students attending the second grade in Department of Digital Media Design at the Ling Tung University in Taiwan. Thus, the number of samples for the experimental group was *N* = 52, and the number of samples in the control group was *N* = 48. The first group was the experimental group, where we integrated our newly developed MAR design system into our curriculum of interior design learning. The control group was not exposed to our new way of experimental teaching as part of their curriculum. The interior design knowledge test is presented in Appendix B of this publication. Each learning theme consists of different topics, and each of these are evaluated. Students are tested through an examination before and after our experience on their knowledge in interior design.

### 4.2. Knowledge Test Questionnaire Design

Our questionnaire was designed to evaluate our new way of experimental teaching in terms of learning motivation, following the theories of learning motivation presented in the literature review section of this paper on the example of an interior design course. The full questionnaire can be found in Appendix A of this publication. The questionnaire was conducted with the control- and experimental group as main subjects of the study at the beginning and at the end of the course. Both the experimental- and the control group filled out the learning effectiveness questionnaire. The different questionnaires were created and collected using Google Forms, and IBM’s Statistical Product and Service Solutions (SPSS) was the tool of our choice for processing statistical results of the study. We conducted an independent sample *t*-test for the pre-experiment data processing and analyzed the difference of both groups in terms of pre-test scores to understand the homogeneity of both groups of students. No significant levels of the *t*-test show that both groups are homogenous, which indicates that the subsequent test can be used to verify if the subsequent tests can be used to verify whether our new way of experimental teaching would improve the effect of utilizing MAR in teaching for interior design.

The pre- and post-experiment questionnaires tested the ability of students on their degree of cognitive understanding of various aspects of interior design layout plans, the design symbols used in interior design, and the ability to recognize and interpret interior design plans. The questionnaires were designed in a way, that it is possible to gain understanding in the improvement of the students’ knowledge after and before teaching activities. These included understanding of interior design structures, dimensioning of designs, different layouts, flow, and utilized design symbols. The questionnaires covered all these five aspects, and measured the knowledge of students accordingly:

1. Students understand the structure of interior design layout and plans;
2. Students understand the dimensioning of interior design layout and plans;
3. Students understand the flow of interior design layout and plans;
4. Students understand the layout functions in interior design layout and plans;
5. Students understand design symbols and placement of design objects as e.g., sofa, TV, bathroom, closet, and other furniture components.

### 4.3. Design of the Evaluation of Student’s Motivation through the ARCS Model

**Figure 5:**
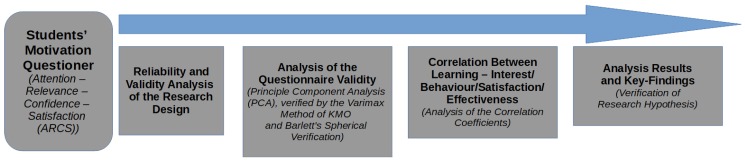
Various stages of testing the student’s motivation through a quantitative analysis.

The ARCS model parameters were evaluated through a questionnaire. Each of the facets of the model were evaluated through a different set of questions. Their analysis stages are presented in Figure 5. Based on the ARCS model (see Table 1) elements and definitions, the proposed evaluation scale includes needs to include the following four components:

- ATTENTION—learning interest (5 questions),
- RELEVANCE—textbook design and teaching methods (5 questions),
- CONFIDENCE—learning behavior performance (confidence 5 questions),
- SATISFACTION—learning satisfaction (5 questions).

The definition and design description of the four components can be found in the following [6]:

1. Learning interest is referred to an intrinsic tendency, as an individual concentrate on a certain activity. It is also a part of the learner’s personality. In general, learners will take more effort and time for an activity that they are interested in and from which they are obtaining satisfaction [31]. Following the same patter, learning interest refers to students investing their efforts and time to obtain satisfaction while experiencing a learning progress.
2. Evaluation of the design of textbooks, teaching materials, and methods to identify the level of student interest in MAR-based learning after letting the students familiarize themselves with the subject matter of interior design courses, and the new teaching technology.
3. Learning behavior to measure whether students have improved their self-confidence after using MAR.
4. Studying the satisfaction of the facet “learning” therefore means to understand whether students agree to use MAR, and then furthermore to evaluate the degree of likes and dislikes of MAR, and whether there is a willingness to use it again in future learning sessions.

**Figure 6:**
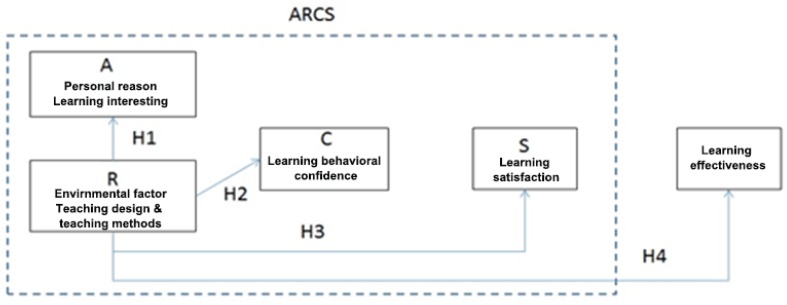
ARCS learning effectiveness statistical model, its parameters, and hypothesis.

Each facet of the questionnaire was based on the Likert’s five-point scale. Students entered the scores into the questionnaire according to the student’s learning status on a scale between 1 and 5 respectively. The different options were: “strongly agree” 5, “agree to “4, “average” 3, “disagree” 2, “strongly disagree” 1. The higher the option score was, the better the particular ability was in the evaluation. If the option score is lower, the ability of students to cope with a particular aspect is poor. The quantitative empirical model is shown in a structured from in Figure 6.

The different hypothesis underlying our research are listed below:

**Hypothesis 1** **(H1).** *“The use of MAR in teaching design and methods positively affect students’ **interest** in learning”.*

**Hypothesis 2** **(H2).** *“The use of MAR in teaching design and methods positively affect students’ **learning behavior**”.*

**Hypothesis 3** **(H3).** *“The use of MAR in teaching design and methods positively affect students’ **learning satisfaction**”.*

**Hypothesis 4** **(H4).** *“The use of MAR in teaching design and methods positively affects student’s **learning outcomes**”.*

## 5. Testing Student’s Subject Knowledge Prior to and after the Experiments

The experimental teaching course took place between 20 September 2018 and 13 January 2019 for both, the experimental group using our new MAR-based teaching method and our control group using traditional ways of teaching. We conducted a knowledge-based test (examination) conducted by the teacher of students before the starting of the teaching period between 5 September 2018 and 20 September 2018 for five days. We also conducted a post-experiment knowledge-based test during the third phase of the experiment, which was conducted between 15 January and 25 January 2019 for 10 days. A total of 52 answers were collected from the experimental group, and 48 of the control group, leading to a total of 100 valid questionnaires. After questionnaire data collection, these were statistically analyzed:

1. Analysis of average number and standard deviation of the test results before and after the analysis of the experimental group and the control group by the independent sample t.
2. Evaluation of test scores for learning outcomes’ five indicators were evaluated for single-factor covariance analysis to examine if the two groups of students were homogenous.
3. Two-way learning results and one-way analysis of variance (ANOVA) to determine whether the different teaching methods affect difference in learning outcomes.
4. Correlation analysis of the ARCS learning motivation model for the research model.
5. Regression analysis to validate research models and research hypotheses.

### 5.1. Evaluation of Learning Performance for Test and Control Group (Independent Sample t-Test Analysis)

The curriculum knowledge test for interior design included the following test for the following five variables of student’s knowledge: (1) structure, (2) dimension, (3) flow, (4) layout, and (5) symbol index. Several of these were obtained after the test. It is known from the data in Table 2, that shows the results of the pre-test for both, experimental and control group, that the experimental results for both groups are quite close. The post-test scores of the experimental group were higher than the control group. In order to understand whether the difference between the two groups reached a statistically significant level, we then tested the homogeneity of the regression coefficients within the group to confirm whether the regression lines for both groups were parallel. This indicates, that both groups are comparable, and there is no statistical difference between both groups. Thus, Table 3 is an overall description of the statistics for both groups but does not determine the differences between both groups. This requires additional analysis, which is described within the following sections of the article.

### 5.2. Determination of the Learning Ability of both Test- and Control Groups

To follow up statistical analysis presented in Table 2, we first needed to determine if the learning ability of each group (experimental vs. control group) is similar or the same. We tested both groups in parallel. In order to understand whether the difference between the two groups was of statistical significance, we used the homogeneity test of the regression coefficient within the group to examine whether there was any interaction between the pretest scores of the two groups. This was to confirm whether learning abilities of both groups were the same. This could be tested through verifying if regression lines within the group are parallel. We selected the one-way ANOVA to test whether the two groups (experimental and control group) were homogenous. The results of the verification are shown in Table 4. These tests indicated if the learning ability of both groups was the same. Thus, the pre-test scores of both groups did not reach any significance via the homogeny test, which satisfied the parallel tests. It indicated that the pre-test of the two groups had no interaction, and both groups were statistically independent from each other.

It can be seen from Table 3 that the difference of experimental group and the control group in the categories “structure” (*F* = 0.043, *p* = 0.836 > 0.05), “dimension” (*F* = 0.657, *p* = 0.42 > 0.05), and “flow” (*F* = 0.158, *p* = 0.51 > 0.05), “layout” (*F* = 0.237, *p* = 0.627 > 0.05), “symbol” (*F* = 0.155, *p* = 0.695 > 0.05) are not significant. This satisfies the parallel check, indicating, that both groups were homogenous. It is consistent with the homogeneity test of the regression coefficient within the group. It can be determined that the exclusion of different classes will affect the pre-test results. Therefore, this study can further adopt a single-factor covariate analysis: post-test scores can be based on variables to include covariates for *F*-tests.

### 5.3. Evaluation of Learning Outcomes (Single Factor Covariate Analysis)

In the previous part of the statistical analysis, the results were used as covariates. The post-test scores of both groups were used as the variables for the effectiveness check. The independent sample single factor covariate analysis was performed. The statistical analysis showed a significant level setting of 0.05 (within a 95% confidence interval). As can be seen from Table 5, the results of single factor covariation analysis showed that the experimental group and the control group had significant differences in “structure” (*F* = 17.828, *p* < 0.001), “flow” (*F* = 84.455.864, *p* < 0.001), “layout” (*F* = 27.030, *p* < 0.001) “symbol” (*F* = 3.357, *p* < 0.005). No significant difference can be seen for the category “dimension” (*F* = 2.258, *p* > 0.05).

Significant differences can be seen for the category and there was no significant difference; the “symbol” learning theme after MAR experiment teaching did not reach a significant difference.

According to the above test results, the experimental and control group are in the categories “structure”, “dimension”, “flow”, “layout” and “symbol” met the homogeneity test of the regression coefficient in the group by using the single factor covariate as analysis basis. The results of the single factor covariation analysis showed that the experimental group and the control group had significant differences in the categories “structure”, “flow”, “layout” and “symbol” indices. After adjusting the adjusted averages, it was found that the experimental group was superior to the control group, while the category “dimension” did not show a significant difference. This result can be used to demonstrate the difference in learning outcomes between both groups. The experimental group of students using the MAR application as a learning aid achieved significant better learning outcomes than those in the control group not using the MAR application.

## 6. Evaluation of Students’ Motivation through the ARCS Questionnaire

### 6.1. Descriptive Statistic and Reliability of the Analysis

Firstly, the descriptive statistical data from the questionnaire (Appendix A) which was collected from the experimental group show that the average value of each component of the ARCS model (Figure 6) is the average of all the questionnaire scores of the standard deviation, and the average result of each component in this study. As shown in Table 6, the average number of each component shows that the experimental group students have a positive and satisfactory evaluation of each component of ARCS after experimental teaching. This indicates that the experimental group students are affirmed by the MAR teaching materials which are provided by the teacher. It also stimulates positive learning interest, self-confidence, and learning satisfaction:

We evaluated the reliability and validity of our analysis and approach. To evaluate the reliability levels of the study, we utilized the Cronbach value, which indicates with value of *α* < 0.35 low confidence; values between 0.35 < *α* < 0.70 medium confidence, and an *α* value >0.7 a high reliability. The reliability of the results of the statistical analysis of each facet were 0.929 for learning interest; 0.782 for textbook design and teaching method; 0.967 for learning behaviour and 0.900 for learning satisfaction. The Cronbach α reliability levels of our study are above 0.7. Thus, overall the reliability of the questionnaire in this study shows a high confidence coefficient. Within the acceptable range, it has an inherent convergence consistency, indicating that it has a certain level of reliability.

Since the units of the variable measurements are the same in this study, as they are based on the Likert five-point scale, the reliability can be measured by Cronbach’s *α* value. More generally, Cronbach’s *α* value <0.35 shows a low confidence, 0.35 < *α* < 0.70 a medium confidence, and α value greater than 0.7 means that the reliability is quite high. It represents the evaluation of the reliability of the whole scale. The questions in this questionnaire show a high homogeneity and conform to the standard [9]. 

### 6.2. Analysis of the Questionnaire Validity

In this study, SPSS was used for confirmatory factor analysis (ES) to determine the facet validity of the questionnaire. The factor extraction was performed using principal component analysis (PCA) and were verified by the varimax method of KMO (Kaiser-Meyer-Olkin measure of sampling adequacy) and Barlett’s spherical verification. The KMO sampling suitability was 0.516. The square value of the spherical check is 523.029, which is significant, and an indication that the questionnaire question is applicable for a factor analysis. Through factor analysis, the previously set facet questions are automatically aggregated into a single facet scale.

The measurement items of each facet can be converted into a single factor facet (the feature value of each facet needs to be greater than 1), which has a certain level of validity. The decision on the number of factors is mainly based on the principle of the size of the special value. The characteristic value represents the total variation that can be explained by a certain factor. The larger the value, the stronger of the explanatory power are representing the factor.

In general, the eigenvalues need to be greater than the one to be considered as a factor. Through the PCA [32] and the maximum revolving method, our research design has extracted four components. The square of the rotation axis, and the load show a “learning behavior” value of 4.529; “learning interest” value of 4.125; a “study satisfaction “value of 3.751; and “textbook design” a value of 2.976. The Eigenvalues of the components of the square of the rotation axis, and the load are all greater than one (as shown in Table 7), indicating that the questionnaire has good validity from its structural viewpoint.

### 6.3. Correlation between Learing Interest-Teaching Design-Behaviour-Satisfaction-Effectiveness (Analysis of the Correlation Coefficients)

The study used Pearson correlation analysis to test the hypothesis of the two variables proposed in our statistical model. According to the analysis results, the resulting correlation coefficient matrix between the two variables was analyzed (see Table 6). Based on the empirical results of this study, it was found that:

1. there was a significant positive correlation between the “learning interest” of the experimental group and the MAR “teaching design”, with a correlation coefficient of 0.618;
2. the “learning interest” and “learning behavior” of the experimental group were significantly positively correlated, with a correlation coefficient of 0.617;
3. there is a significant positive correlation between “learning interest” and “learning satisfaction” in the experimental group with a correlation coefficient of 0.694;
4. “learning interest” and “learning” of the experimental group show a significant positive correlation between the results with a correlation coefficient of 0.665.

As shown in Table 8, “teaching design” has a significant positive correlation with the “learning satisfaction” (correlation coefficient of 0.618) and “learning effectiveness” (correlation coefficient of 0.393) of the experimental group. It also shows a correlation coefficient with learning behavior of 0.511 and is significant. We continued by validating Kintsch’s argument discussed in [14,33]: to increase interest in learning, it is necessary to cut in from the two factors of the learner, namely, emotional interest and cognitive interest. Emotional interest theory refers to the material that provides interesting content on the content or theme of the textbook. Cognitive interest is that after the learner understands the subject matter, intrinsic cognitive learning becomes more interesting and affects learning behavior to create good learning performance [14,33].

It is confirmed by our study that there is a positive relationship between learning interest and appealing and interesting design of the content or material of the learning materials. The learner’s cognition of MAR textbooks results in positive learning behaviors in the learning process (i.e., increases the number of learning, etc., and shows satisfaction with the use of MAR). Thus, these alter the effectiveness of students’ learning. From the correlation coefficient analysis results, we can state, that there is a positive correlation (significant existence) between the various components of our research model.

### 6.4. Analysis Results and Key Findings through A Verification of Research Hypothesis

This section conducts the verification of the overall statistical model of our research through a path analysis. Path analysis is a combination of regression analysis. In addition to regression analysis, different functions are combined to form a structured model through a hypothetical research framework. Through this analytical method, this study analyzes the relationships between the causes and effects of the dependent and independent variables (e.g., y = *f*(x)) and analyzes the relationship between direct or indirect effects between variables. Based on this analysis, *R*^2^ is used as the percentage of the total variation of the variable that can be explained by the independent variable and the regression model. We use SPSS to perform the complex regression statistical analysis. We calculate the effect of single or multiple independent variables on the direct influence of variables to obtain the β influence coefficient and determine whether the path exists according to its significance or not. We adjust the *R*^2^ value look at the explanatory power analysis of this research model.

The following is a path analysis for the four research hypotheses in this paper:

**Hypothesis 5** **(H5).** *“The use of MAR in teaching design and methods positively affect students’ interest in learning” (function 1: Learning interest = f {Textbook Design}): according to the ARCS theory proposed by Keller, the factors influencing learning interest are teaching methods and textbook design in environmental factors (referred to as “textbook design” in this study). Among them, β = 0.618, p < 0.001, R^2^ = 0.383), the textbook design of MAR “textbook design” is positively affecting the factors of “learning interest” of the experimental group students. The explanatory power is 38.3%, reaching a significant level, showing the H1 hypothesis is valid.*

**Hypothesis 6** **(H6).** *“The use of MAR in teaching design and methods positively affect students’ learning behavior” (function 2: Learning Behavioral Performance = f {Textbook Design}): after regression statistics, “teaching material design” is one of the factors affecting learning behavior (β = 0.511, p < 0.001 is significant, of which R^2^ = 0.261). It can be seen that the textbook design of MAR “textbook design” is positively affecting the factors of “learning behavior” of the experimental group students and reaches a significant level. The H2 hypothesis is established. It can be seen that the students in the experimental group are still affected by MAR “teaching design” in terms of learning behavior.*

**Hypothesis 7** **(H7).** *“The use of MAR in teaching design and methods positively affect students’ learning satisfaction” (function 3: Learning Satisfaction = f {Textbook Design}): after regression statistics, “teaching material design” is one of the factors affecting learning satisfaction (β = 0.351, p < 0.05 is significant), of which R^2^ = 0.123). It can be seen that the textbook design of MAR “textbook design” is positively affecting the factors of “study satisfaction” of the experimental group students and reaches a significant level. The H3 hypothesis is established. The learning satisfaction of the experimental group students is influenced by MAR “textbook design”.*

**Hypothesis 8** **(H8).** *“The use of MAR in teaching design and methods positively affects student’s learning outcomes” (function 4: Learning effectiveness = f {Textbook Design}): after regression statistics, “teaching material design” is one of the factors affecting learning outcomes (β = 0.393, p < 0.05 is significant, of which R^2^ = 0.155). The textbook design of MAR “textbook design” is positively affecting the factors of “learning effectiveness” of the experimental group students, and it has reached a significant level. The H4 hypothesis is established. The learning outcomes of the experimental group students are influenced by MAR “textbook design”.*

From the results of the above regression analysis, it can be determined that MAR-based teaching materials positively influences student’s learning interest, satisfaction, effectiveness for the group of students that have been utilizing this technology. Students in the control group did not show the same positive influence. Thus, integrating MAR technology into the classroom of interior design lectures helps students in understanding 3D structures, dimensioning, tabular shape flows, interior symbols in final interior design plans before their implementation in a real indoor design environment.

However, AR and the dynamic features it provides to support the interior design process affects the students’ interest in learning indoor design, learning behavior, learning satisfaction, and learning effectiveness. This is supported by the outcomes of our study. Thus, most of the students using our MAR system show a high learning ability to recognize interior symbols and understand interior design layouts and plans. They show an increased capability of imagining these spatially in 3D. During the course of this experiment, students were excited and interested when confronted with the task to read design layouts and plans using AR technology. We strongly believe, that AR has attracted the attention of students and has successfully helped student to recognize different interior design symbols, and have an increased understanding of the structure of interior design layouts and plans.

## 7. Conclusions and Discussions

For this study, we developed an augmented reality display interface on a mobile phone platform, which was utilized to visualize interior design layouts and plans. AR technology enabled the instant display of the spatial composition of interior designs and design artefacts as e.g., furniture accessories over physical interior design plans. This technology was evaluated on its applicability as teaching tool. The utilization of the tool as teaching aid has been examined throughout a complete one-semester interior design course. We investigated whether the tool supports the learning experience, how it can be integrated actively during the curriculum creation phase, and how it improves learning outcomes.

This study uses the ARCS theoretical model proposed by Keller [14] as a research model to verify the effectiveness of the integration of MAR technologies into curriculum designs. We conducted a statistical analysis on two distinct groups of students, one utilizing AR technology and the other one focusing on traditional learning technologies. We tested the reliability and validity of our statistical model, after performing various analysis techniques including a correlation analysis/regression analysis. Our main conclusion is that the application of MAR software in teaching improves learning and teaching effectiveness. Within the following sections we compile the key-findings of our statistical analysis and experiments and compare these to our research questions.

### 7.1. Keller’s ARCS Model of Motivational Instructional Design Theories are Applicable in the Analysis of New Digial Learning Technologies in Teaching and Provide a Useful Analysis Tool

**Figure 7:**
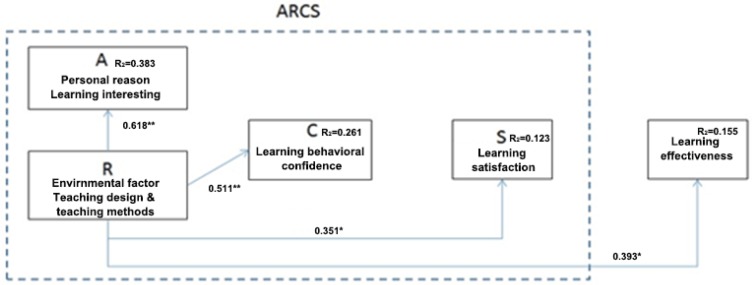
Overall regression analysis of this study. The results of the regression analysis of this study are shown through arrows between the different factors. If the regression analysis following an arrow is of significance, the normalized coefficient data will have an asterisk (*). This indicates that the causal relationship has a direct effect, which can be confirmed by the letter. Otherwise, if the normalized coefficient data is not significant, there will be no asterisk, indicating that there is no direct effect, and the arrow segment does not exist.

The ARCS model integrates many known learning motivational theories. However, the theories’ core concept lies in the provision of a well-systematized approach of instructional and learning designs. Thus, the design of teaching materials is more in-line with participatory learning designs and provides interactivity to inspire learners. As the design of teaching materials is main contributing factor to attract students’ attention and maintain interest in the learning process. Without the considerable attention of students, and without their interest in learning content or learning methods, the learning outcome will be poor. Our study focused on the aspect of introducing new AR technology into the learning process and provides some guidelines how teachers will be able to integrate various methods for interior design courses. In order to prove that MAR-based textbooks is a feasible method supporting Keller’s theory, we used regression analysis (see Section 6.4) to verify that MAR textbooks did have a positive impact on learning interest, learning confidence, learning satisfaction, and student performance (see Figure 7). By this, we effectively proved that Keller’s theory can be combined with the application of novel innovative digital technology into teaching practise for interior design courses. Thus, Keller’s theories in motivational instructional design theories are applicable for new digital learning technologies as e.g., MAR is.

### 7.2. Innovative Technologies such as Mobile Augmented Reality (MAR) Teaching Materials Increase Student’s Interest in Learning

In educational research, it is known that the use of new innovative technologies lets new innovative teaching modalities emerge and supports user (student) centred learning designs. This makes the use of new technologies an attractive solution to efficiently increase learning experiences. Similarly, in common design research theory, as e.g., discussed in Dieter Rams’ works [34], good designs are innovative, and today’s possibilities for innovation are not, by any means, exhausted. Technological development is always offering new opportunities for innovative designs. But innovative design always develops in tandem with innovative technology, and can never be an end in itself [34]. Our results confirm these claims, and the use of new innovative technologies such as AR-based teaching materials improve the students’ interest in learning. As we expected at the beginning of the study, our proposed research hypothesis Hypothesis 5 “the use of MAR in teaching design and methods positively affect students’ *interest* in learning” has statistically been verified. One of the main key restrictions is whether AR content meets the needs of teaching content to be delivered to students or not, which should be part of the discussion when developing the overall learning design. While our study clearly indicates that a MAR system supports the instructional design-centered needs of students, an overall restriction is the question of whether the AR based material can attract the interest and focus of the learning for the teaching subject. If this cannot be achieved, the learning effect will greatly be reduced. Thus, the learning designer needs to evaluate weather AR is useful in teaching students in a particular context.

### 7.3. MAR Teaching Materials Increase Students’ Motivation, in Particular Learning Behavior and Satisfaction

As we have been concluding previously, learning interest is enhanced through the application of MAR-based teaching designs and methods. AR based interactivity and instant viewing of design alternatives enhances learning interest. Students are fully immersed in the simulated interior design situation, leading to an enhancement in their satisfaction and overall learning process. Our study confirms that the students’ ability to understand different interior design strategies utilizing MAR technology improves, and MAR technology improves students’ motivation, attention, and overall increases the performance of learning behaviour. Thus, an AR-based textbook design, has positive effect on learning behaviour, learning satisfaction, and learning effectiveness. This has been proven by the linear regression analysis that we have been conducting and conforms our hypothesis Hypothesis 6 “the use of MAR in teaching design and methods positively affects students’ learning behaviour”, and Hypothesis 7 “the use of MAR in teaching design and methods positively affects students’ learning satisfaction”.

### 7.4. AR Technology Provides New Learning Experiences in terms of Understanding Learning Content Understanding Spontaneous Learning Behaviours and Increased Learning Performance

We also would like to underline several other positive aspects that emerge based on our results: first, students can self-study, thus they can guide themselves through improving their skills through repeating different design approaches; second, through repeated practice in creating interior design layouts, playing with design alternatives e.g., such as placing furniture and accessories differently, and being presented with a 3D visualisation in the instant of the final layouts, students have an increase sense of imagination as to what layouts look like when physically implemented; third, student develop spontaneous learning behaviours by being exposed to instant visualisations of their interactive inputs. This relates to indoor space settings, furniture placements, accessories in space, and auxiliary functions such as lighting and the viewer’s orientation. These can be adjusted arbitrarily, and students develop a playful way of spontaneous learning behaviours. This behavior supports the ability to understand and inspire interior designs, the creation of design alternatives, and enhances the overall learning satisfaction. It is obvious, that this leads to better learning outcomes [35]. Thus, we see our initial hypothesis Hypothesis 8 “the use of MAR in teaching design and methods positively affects the students’ learning outcomes”.

### 7.5. Increased Reinforcement of Students’ Motivation through AR Technologies

Learning satisfaction is an evaluation of students’ learning outcomes and is an important factor in learning motivation. Empirical research has shown that contextualized learning is possible by integrating AR technology and its interactive functions. These allow students to experience simulated situations of interior design alternatives, and to apply the knowledge and skills that they have learned. This enhances learning satisfaction and reinforce student motivation. This study clearly demonstrates that AR technology applied in teaching designs is feasible and effective through a quantitative verification results of Keller’s ARCS motivational design model. 

### 7.6. Increased Student Subject Knowledge in Interior Design

Through our knowledge tests, which evaluated different aspects of knowledge in interior design, we have also shown that the student’s subject knowledge improves through the introduction of MAR technologies. Interestingly, while students improved in understanding facets such as structure, flow, layout, and design symbols better, the knowledge around dimensioning did not significantly improve. This might be explained by the nature of AR, which combines the strength of a physical representation of layout plans overlaid by a virtual scene. Through this, the strength of AR lies in the added value through providing interactivity, and graphics overlays. The ARCS motivation model can provide educators with design strategies for identifying and understanding students’ motivational needs to promote learning motivation and effectively improve student learning and performance. After the experimental verification of this research, the students as a whole affirmed the systematic teaching design of this research MAR (see Table 6), and it achieved the following criteria: (1) attracting attention, (2) the teaching materials can be appropriately linked to the student’s learning experience, so as to arouse the students’ learning motivation, (3) build confidence (4) satisfaction. Keller [13] emphasized that the application of ARCS motivation model A, R, C, S four factors are interlinked, and the positive direction of each link will definitely make the student’s learning a virtuous circle. This in particular is of relevance for interior design students, as they need to be aware about spaces, and how these spaces can be created. Thus, pre-imagination of possible design configurations, layouts, and how these might look in reality is a major required skill set. This also includes the evaluation of different design alternatives and provides the best possible interior design solution.

Not only did we prove that AR technology provides a useful tool for interior design students, we have also been developing a method for evaluating and verifying the use of new innovative technologies in a teaching setting following Keller’s motivational learning design model. The method is exhaustively described in the method section of this article, and can be used for similar experimental settings as part of analysis the use of technology other than AR. To conclude this study, we have proved that AR technology in teaching improves students’ learning experience. We also developed an efficient method in evaluating similar kinds of learning situations. The method is easily applicable for similar kinds of experiments.

## Figures and Tables

**Table 1:** Four elements and definitions of Attention, Relevance, Confidence, and Satisfaction (ARCS) model, following John Keller’s definitions [14].

Element	Definition	Variable	Purpose
**A** **Attention**	Arouse the interest of students, maintain the attention of students, and stimulate the curiosity of students.	Learning interest	1. Use the learning materials provided by mobile augmented reality (MAR) to arouse students’ interest in learning. 2. Observe students’ curiosity about the subject of learning, use time, and increase their concentration.
**R** **Relevance**	Students develop relevant personal recognition based on the learning of new textbooks and past experience.	Teaching design and method	1. Whether students are immersed in MAR to provide interior design learning. 2. Does the student affirm the learning experience with MAR as the teaching material?
**C** **Confidence**	Arouse students’ expectations of success and positive attitudes towards students to help students build self-confidence.	Learning behavior	1. Students must use MAR to master the steps of learning and be useful for learning. 2. Students use the confidence and concentration gained in MAR.
**S** **Satisfaction**	Students’ satisfaction and sense of accomplishment in the experience and results of learning will enhance their self-learning effectiveness.	Learning satisfaction	1. Use MAR to let students start self-learning, gain greater satisfaction and sense of accomplishment, and produce lasting learning interest.

**Table 2:** Software run-through and comparison between experimental AR-based and traditional teaching.

Traditional Teaching Methods	AR-Based Teaching Approach
**1st Topic Module** **Teacher:** Assignment Questions and Explanation of Design Cases: Structure case study + design concept guide. **Students:** Structure design sketch + formal draft blueprint drawing. Design formal blueprint sketch + formal draft model design. 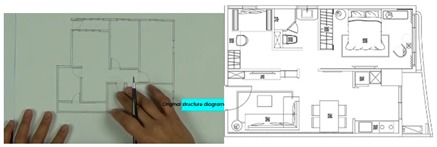 **Teacher:** Review and comment for the first theme unit.	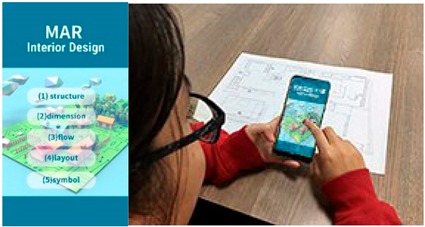 **Teacher:** Assignment Questions and Explanation of Design Cases. Structure case study + design concept guide. **Note:** (1) The teaching subject content is the same as traditional teaching, but AR-assisted learning is used in the structure case analysis. (2) Interior structure examples study with AR. **Students:** Study empty interior structure and learn the symbol in the right place with AR.
**2nd Topic Module** **Teacher:** Assignment Questions and Explanation of Design Cases: Dimension case study + design concept guide. **Students:** Dimension design sketch + formal draft blueprint drawing. Design formal blueprint sketch + formal draft model design. 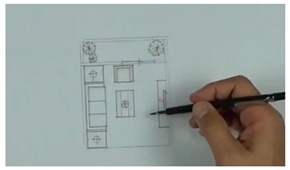 **Teacher:** Review and comment for the second theme unit.	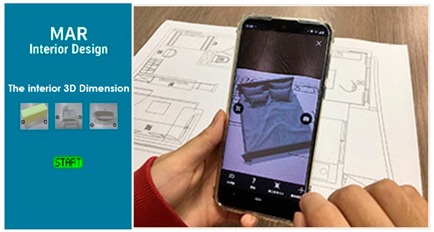 **Teacher:** Assignment Questions and Explanation of Design Cases: Dimension case study + design concept guide. **Students:** Learn the interior 3D dimensions with AR. The 3D imagination model appears on the screen from the system library.
**3rd Topic Module** **Teacher:** Assignment Questions and Explanation of Design Cases: Flow case study + design concept guide. **Students:** Flow design sketch + formal draft blueprint drawing. Design formal blueprint sketch + formal draft model design. **Teacher:** Review and comment for the third theme unit.	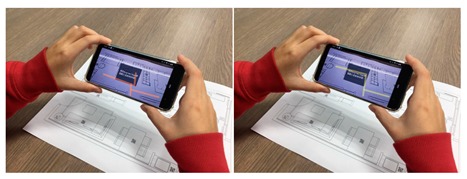 **Teacher:** Assignment Questions and Explanation of Design Cases: Flow case study + design concept guide. **Students:** Students use AR function for flow case study.
**4th Topic Module** **Teacher:** Assignment Questions and Explanation of Design Cases: Layout case study + design concept guide. **Students:** Layout design sketch + formal draft blueprint drawing. Design formal blueprint sketch + formal draft model design. 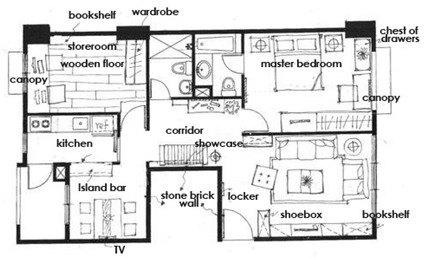 **Teacher:** Review and comment for the fourth theme unit.	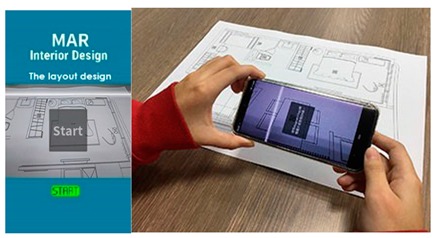 **Teacher:** Assignment Questions and Explanation of Design Cases: Layout case study + design concept guide. 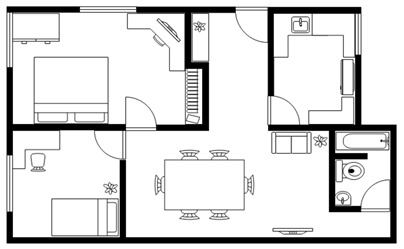 **Students:** Use of AR functions to understand layout design cases.
**5th Topic Module** **Teacher:** Assignment Questions and Explanation of Design Cases: Symbol case study + design concept guide. **Students:** Symbol design sketch + formal draft blueprint drawing. Design formal blueprint sketch + formal draft model design. **Teacher:** Review and comment for the fifth theme unit.	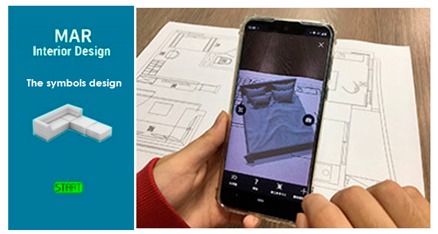 **Students:** Learn the different between 3D dimensions and flat symbols with AR.

**Table 3:** The control- and the experimental group’s knowledge measured before and after the interior design test conducted by the teacher. It illustrates the overall learning performance of both groups, but further statistic evaluation is required to identify the performance of each individual test group.

Test Item	Group	Number of People	Pre-Test Average	Pre-Test Standard Deviation	Post-Measurement Average	Post-Test Standard Deviation
structure	Test group	52	13.7308	2.12469	16.9423	1.88298
	Control group	48	13.8333	2.78547	15.1458	2.394
dimension	Test group	52	13.2308	2.20174	16.1346	1.69230
	Control group	48	13.6250	2.65478	15.5208	2.36094
flow	Test group	52	9.3077	1.66319	17.0000	1.83645
	Control group	48	9.4375	1.59662	13.3542	2.12873
layout	Test group	52	12.2692	2.89770	16.7885	2.32072
	Control group	48	12.5625	3.12101	13.9167	3.16788
symbol	Test group	52	11.9231	2.65571	16.0385	1.94998
	Control group	48	11.7083	2.79786	14.4792	3.11475

**Table 4:** Summary of testing if the learning ability of both groups is the same, thus, if they are comparable. This test was conducted in the pre-test stage of the experiment to test if both groups are comparable and show same learning ability. Results show no significant difference between both groups.

Test Item	Source	SS	df	MS	*F* Value	Variability Homogeneity Test Significance
structure	Both groups in pre-test stage	0.263	1	0.263	0.043	0.836
		594.897	98	6.070		
dimension	Both groups in pre-test stage	3.879	1	3.879	0.657	0.420
		395.839	98	5.903		
flow	Both groups in pre-test stage	0.421	1	0.421	0.158	0.692
		260.889	98	2.662		
layout	Both groups in pre-test stage	2.147	1	2.147	0.237	0.627
		886.043	98	9.041		
symbol	Both groups in pre-test stage	1.151	1	1.151	0.155	0.695
	Both groups in pre-test stage	727.609	98	7.425		

*p* > 0.05.

**Table 5:** One-way analysis of variance (ANOVA) summary table for two groups of learning outcomes.

Test Item	Source	SS	df	MS	*F* value	Significance
**Structure**	Difference between groups	80.554	1	80.554	17.828	0.000 ***
		442.806	98	4.518		
**Dimension**	Difference between groups	9.403	1	9.403	2.258	0.136
		408.073	98	4.164		
**Flow**	Difference between groups	331.771	1	331.771	84.455	0.000 ***
		384.979	98	3.928		
**Layout**	Difference between groups	205.850	1	205.850	27.030	0.000 ***
		746.340	98	7.616		
**Symbol**	Difference between groups	60.688	1	60.688	9.151	0.003 **
		649.902	98	6.632		

* *p* < 0.05, ** *p* < 0.01, *** *p* < 0.001.

**Table 6:** Descriptive statistics of the ARCS model components for the experimental group.

Components	Number	Mean	Standard Deviation
Learning interest	52	4.5808	0.20679
Teaching design	52	4.5269	0.17277
Learning behavior	52	4.5462	0.19449
Learning satisfaction	52	4.5769	0.20447
Learning performance	52	4.6462	0.17763
Valid N (excluded completely)	52		

**Table 7:** Factor analysis—rotation sums of squared loadings.

Total Variance Explained						
	Initial Eigenvalue			Rotation Sums of Squared Loadings		
Ingredients	Sum	Variance%	Cumulative	Sum	Variance%	Cumulative%
Learning behavioral	5.594	27.972	27.972	4.529	22.647	22.647
Learning interesting	4.410	22.052	50.024	4.125	20.627	43.274
Learning satisfaction	2.986	14.932	64.955	3.751	18.754	62.027
Teaching design	2.391	11.953	76.908	2.976	14.881	76.908
Extraction method: principal component analysis						

**Table 8:** Correlation analysis.

		Learning Interest	Teaching Design	Learning Behavior	Learning Satisfaction	Learning Effectiveness
Learning interest	Pearson Correlation	1	0.618 (**)	0.617 (**)	0.694 (**)	0.665 (**)
	Significance (two-tailed)		0.000	0.000	0.000	0.000
Teaching design	Pearson Correlation	0.618 (**)	1	0.511 (**)	0.351 (*)	0.393 (**)
	Significance (two-tailed)	0.000		0.000	0.011	0.004
Learning behavior	Pearson Correlation	0.617 (**)	0.511 (**)	1	0.501 (**)	0.618 (**)
	Significance (two-tailed)	0.000	0.000		0.000	0.000
Learning satisfaction	Pearson Correlation	0.694 (**)	0.351 (*)	0.501 (**)	1	0.591 (**)
	Significance (two-tailed)	0.000	0.011	0.000		0.000
Learning effectiveness	Pearson Correlation	0.665 (**)	0.393 (**)	0.618 (**)	0.591 (**)	1
	Significance (two-tailed)	0.000	0.004	0.000	0.000	

* When the significant level is 0.05 (two-tailed), the correlation is significant. ** At a significant level of 0.001 (two-tailed), the correlation is significant. Total number of exclusions = 52.

## Appendix A. Student Questionnaire Questions

### Personal Reason- Learning Interest

1. I think that learning interior design is interesting in the structure theme.
2. I think that learning interior design is interesting in the dimension theme.
3. I think that learning interior design is interesting in the flow theme.
4. I think that learning interior design is interesting in the layout theme.
5. I think that learning interior design is interesting in the symbol theme.

### Learning Attitude for MAR Materials Design

1. I can skillfully use the contents of the structural theme provided by MAR and feel practical.
2. I can skillfully use the contents of the size theme provided by MAR and feel practical.
3. I can skillfully use the content of the process theme provided by MAR and feel practical.
4. I can skillfully use the contents of the layout theme provided by MAR and feel practical.
5. I can skillfully use the contents of the symbol theme provided by MAR and feel practical.

### Learning Behavioral Confidence

1. I have confidence in learning the structure theme.
2. I have confidence in learning the dimension theme.
3. I have confidence in learning the flow theme.
4. I have confidence in learning the layout theme.
5. I have confidence in learning the symbol theme.

### Learning Satisfaction

1. I am satisfied with learning the structure theme.
2. I am satisfied with learning the dimension theme.
3. I am satisfied with learning the flow theme.
4. I am satisfied with learning the layout theme.
5. I am satisfied with learning the symbol theme.

## Appendix B

### Interior Design Learning Evaluation Table

**Table:**

Course Title	Design Exhibition and Marketing	ID	xxx
		Date	
Unit	□ pre-test ▓ post-test	Evaluator	
Design Exhibition Learning Evaluated Constructs		Evaluated factor explanation	Grade
Structure (20%)	1. 3D Structure Learning		
	2. Flat and 3D image translation Learning		
	3. Interior Space Structure Learning		
Dimension (20%)	1. Interior Dimension		
	2. Furniture Dimension		
	3. Aisle Dimension		
Flow (20%)	1. Bag Shape Flow		
	2. Tubular Shape Flow		
Layout (20%)	1. Against the Wall Layout		
	2. Layout Middle Layout		
	3. Compartment Layout		
Symbol (20%)	1. Interior Furniture Symbol		
	2. Interior Electricity Symbol		
	3. Interior Home Appliance Symbol		
